# A Rare Catastrophe: Three Cases of Aortic Root Dehiscence after Surgery

**DOI:** 10.21470/1678-9741-2020-0679

**Published:** 2023

**Authors:** Taner İyigün, Barış Timur, Timuçin Aksu

**Affiliations:** 1 Department of Cardiovascular Surgery, İstanbul Mehmet Akif Ersoy Thoracic and Cardiovascular Surgery Research and Training Hospital, Istanbul, Turkey.

**Keywords:** Aortic Diseases, Ascending Aorta, Dehiscence, Cardiac Surgery, Rupture, Spontaneous

## Abstract

Ascending aortic pathologies may be life-threatening. Postoperative aortic root dehiscence is a very rare but extremely dangerous complication with a high mortality rate, and redo surgery is mandatory due to high risk of spontaneous rupture. We present three cases that had undergone Bentall procedure and had postoperative aortic root dehiscence. One of the patients presented with hemiplegia caused by septic embolus while the others had mild symptoms. Dr. Yakut’s modified Bentall procedure, the flanged technique, was performed for each patient in redo surgery. Two patients were successfully discharged from the hospital, but one died due to intracranial hemorrhage and multiple organ failure.

## INTRODUCTION

Ascending aortic pathologies may be life-threatening due to rupture or dissection. Acute untreated aortic dissection has a mortality rate up to 50% within 48 hours. Emergency surgery statistically significantly decreases the mortality rate. Elective cases have lower (3-5%) mortality rates compared with emergency cases (15-26%)^[1]^. Bentall and De Bono first described the procedure for ascending aortic pathologies — that include aortic valve and sinuses of Valsalva pathologies — in 1968^[2]^. Bentall-De Bono procedure is an effective way to treat dilated ascending aorta with concomitant aortic root and aortic valve pathologies. Aortic root dehiscence and pseudoaneurysm formation following a surgery is a rare but extremely dangerous complication. The most common cause for graft dehiscence in these patients is infection, while surgical technical errors and recurrent aneurysmal dilatation are the other causes^[3]^. Most common sites of dehiscence were the distal suture line, proximal valvular sutures, and coronary button anastomosis sites^[4]^. Mortality rate for the patients complicated by aortic root dehiscence remains noticeably high even if they had the chance of emergency redo surgery^[5]^. Here we present three cases of postoperative aortic root dehiscence.

### Case 1

**Fig. 1:**
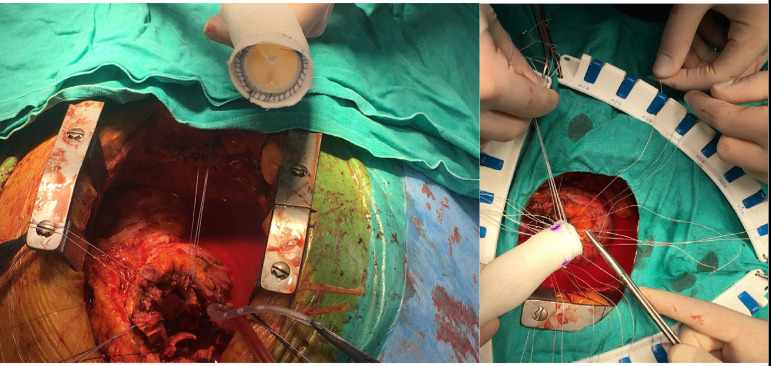
Preparation of Dr. Yakut’s flanged Bentall graft.

A 51-year-old male patient with diagnosis of type 1 aortic dissection had rheumatoid arthritis. Echocardiography revealed dissection flap with moderate aortic valve insufficiency. Contrasted computed tomography (CT) proved the diagnosis of acute type 1 aortic dissection. He was successfully discharged at postoperative 12^th^ day of Bentall procedure. Six months later, the patient was admitted to the emergency department suffering from dyspnea. Contrasted CT revealed type B dissection with periaortic hematoma. He was followed with medical therapy as he refused the surgery, but, unfortunately, he was transferred to the intensive care unit (ICU) in the fourth day of hospitalisation and undergone surgery. Modified Bentall procedure, the flanged technique, with mechanical aortic conduit was used for the patient whose aortic root was dehiscent by the proximal valvular sutures^[6]^ (Figure 1). Aortic root was also strengthened using bovine pericardium. Postoperative period was complicated by subdural hematoma, and the patient died four months after the surgery with multiorgan failure.

### Case 2

**Fig. 2:**
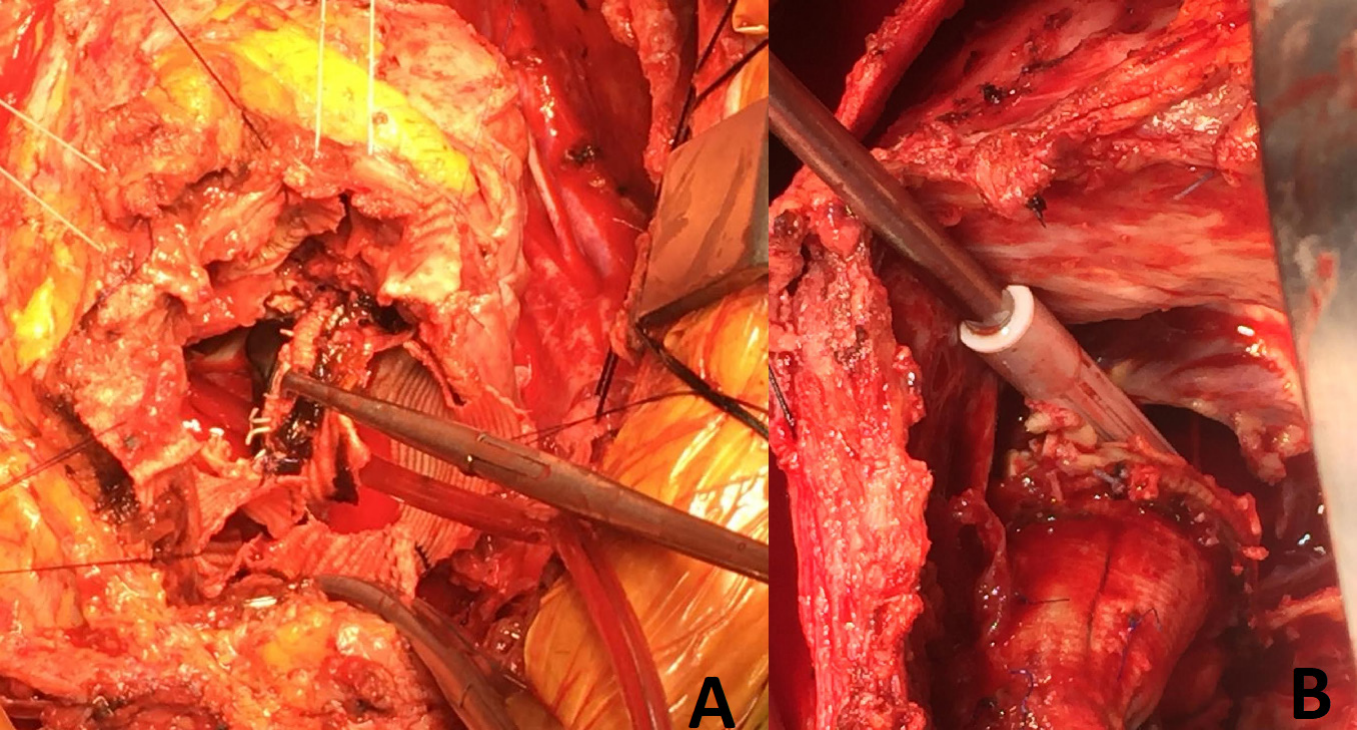
A, B) Preoperative images of the patients’ dehiscenced aortic root.

A 45-year-old male patient with Marfanoid stature was admitted to our clinic with occasional chest and back pain. Contrasted CT and transthoracic echocardiography were performed. The ascending aorta and sinuses of Valsalva were 51 mm and 59 mm in diameter, respectively, with moderate aortic stenosis. After emergency preoperative follow-up, the patient underwent Bentall procedure. On the 7^th^ day, he was discharged from the hospital without any complications. Two weeks after the surgery, the patient was admitted to the hospital with fever, dyspnea, and fatigue. Blood count and biochemical markers were within normal ranges, there was nothing remarkable in urine and blood cultures, and echocardiography was normal. At the 10^th^ day of admission, with prophylactic antibiotic therapy, his general medical condition deteriorated, which was accompanied by tachycardia and hypotension. Leak from aortic root was diagnosed by transesophageal echocardiography, and hematoma around ascending aorta was shown by contrasted thorax CT (Figure 2A). Emergency redo Bentall procedure, flanged technique, with mechanical aortic conduit was performed^[6]^. Aortic root was strengthened using bovine pericardium. The patient was discharged at the postoperative 19^th^ day without any other complications.

### Case 3

A 69-year-old male patient with previous diagnosis of diabetes mellitus and hypertension suffering from angina provoked by physical exertion was hospitalised with the diagnosis of ascending aortic aneurysm and severe aortic regurgitation revealed by contrasted CT and transthoracic echocardiography. Atherosclerotic coronary artery disease was also diagnosed during preoperative follow-up. Coronary artery bypass grafting (CABG) (left anterior descending artery, obtuse marginal first and second branches of circumflex artery, and right coronary artery) and Bentall procedure with mechanical aortic conduit were performed. He was extubated seven hours after the surgery, discharged from ICU on the postoperative 1^st^ day, and discharged from the hospital on the postoperative 7^th^ day. Four months after surgery, in another hospital, infective endocarditis of mechanical valve was diagnosed after his admission with left sided hemiplegia caused by septic emboli. The patient was transferred to our hospital and after four days due to increasing thrombocyte count (from 47000 to 90000), and redo CABG and flanged Bentall technique with bioprosthetic aortic conduit were performed^[6]^. Aortic root was strengthened using bovine pericardium. Vegetations were seen on the leaflet (Figure 2B). He was discharged from the hospital on the postoperative 45^th^ day after termination of antibiotic therapy and referred to physical therapy and rehabilitation for hemiplegia.

## DISCUSSION

Aortic root dehiscence is a rare phenomenon. There are some limited series and case reports in the literature regarding pseudoaneurysm of thoracic aorta^[3,4,7-11]^. Due to its insidious onset, diagnosis of the pathology can be challenging. Clinical presentation of aortic root dehiscence may present with various symptoms. Different series and cases on the matter showed numerous symptoms such as heart failure, chest pain, syncope, headache (due to intracranial infarction), fever, dyspnea, and even seizures^[3,7,9-11]^. Symptoms such as heart failure and chest pain may lead the physician to further investigate the aorta and heart. However, nonspecific symptoms such as fever or symptoms of the central nervous system may mislead the physician. It should be kept in mind that patients that had undergone ascending aortic and aortic root surgery should be investigated thoroughly, no matter the symptoms he/she has.

The underlying pathology that causes aortic pseudoaneurysm may vary as well. The most encountered reason was infection of various anatomic structures such as valve, aortic graft, or mediastinum^[3,4,7-10]^. Different types of microorganisms were reported to be isolated from the cultures, such as *Staphylococcus aureus, Enterococcus*, and *Candida*^[3,8-10]^. On the other hand, Gebhard et al.^[11]^ presented a case with high-dose corticosteroid use after the surgery. As a concomitant risk factor, using a prosthetic valved conduit increases the tendency for dehiscence of the aortic root. Moreover, predisposing factors like rheumatoid arthritis, history of prolonged corticosteroid use, and connective tissue disorder may complicate the postoperative period of successfully managed aortic root surgeries.

The time interval and the reason of dehiscence after surgery may differ. There are cases reported up to 17 years after the first operation^[12]^. Infectious and non-infectious causes were present for dehiscence to occur^[7]^.

Brewer et al.^[13]^, Thubrikar et al.^[14]^, and Lansac et al.^[15]^ proved the importance of the dynamic properties of aortic root. Therefore, using a mechanical conduit may disrupt this dynamic feature of the aortic root and ascending aorta and lead to dehiscence. Yakut’s modified surgical technique leading to post-surgical aortic root most similar to physiological root may be the reason of surgical success^[6]^.

## CONCLUSION

Aortic root dehiscence after aortic root surgeries is an extremely rare but mortal complication. Redo surgery is mandatory due to high risk of spontaneous rupture. We prefer and advise the use of flanged Bentall technique for reoperations after root dehiscence, as it provides physiologic motion of the root more than other modified Bentall procedures. The flanged technique may be an effective alternative method for aortic root dehiscence after surgery.

## References

[1] Prakash P, Patni R, Asghar NM, Chan KMJ, Antanas M (2011). Ascending aorta aneurysms: pathophysiology and indications of
surgery. EJ Cardiol Pract.

[2] Bentall H, De Bono A (1968). A technique for complete replacement of the ascending
aorta. Thorax.

[3] Atik FA, Navia JL, Svensson LG, Vega PR, Feng J, Brizzio ME (2006). Surgical treatment of pseudoaneurysm of the thoracic
aorta. J Thorac Cardiovasc Surg.

[4] Niederhäuser U, Künzli A, Genoni M, Vogt P, Lachat M, Turina M (1999). Composite graft replacement of the aortic root: long-term
results, incidence of reoperations. Thorac Cardiovasc Surg.

[5] Mulder EJ, van Bockel JH, Maas J, van den Akker PJ, Hermans J (1998). Morbidity and mortality of reconstructive surgery of noninfected
false aneurysms detected long after aortic prosthetic
reconstruction. Arch Surg.

[6] Yakut C (2001). A new modified Bentall procedure: the flanged
technique. Ann Thorac Surg.

[7] Oh KT, Derose J, Taub C (2016). Fortune or misfortune: asymptomatic, delayed presentation of
complete dehiscence of mechanical aortic valve conduit and
pseudoaneurysm. BMJ Case Rep.

[8] Kannan A, Smith C, Subramanian S, Janardhanan R (2014). A rare case of prosthetic endocarditis and dehiscence in a
mechanical valved conduit. BMJ Case Rep.

[9] Stiver K, Bayram M, Orsinelli D (2010). Aortic root bentall graft disarticulation following repair of
type a aortic dissection. Echocardiography.

[10] Tabakci MM, Yazicioglu MV, Toprak C, Demirel M, Avci A (2016). Collapse of aortic graft through its disarticulation secondary to
periaortic root abscess: an unusual cause of syncope. Echocardiography.

[11] Gebhard C, Biaggi P, Stähli BE, Schwarz U, Felix C, Falk V (2013). Complete graft dehiscence 8 months after repair of acute type A
aortic dissection. Eur Heart J Acute Cardiovasc Care.

[12] Mohammadi S, Bonnet N, Leprince P, Kolsi M, Rama A, Pavie A (2005). Reoperation for false aneurysm of the ascending aorta after its
prosthetic replacement: surgical strategy. Ann Thorac Surg.

[13] Brewer RJ, Deck JD, Capati B, Nolan SP (1976). The dynamic aortic root. Its role in aortic valve function. J Thorac Cardiovasc Surg.

[14] Thubrikar M, Bosher LP, Nolan SP (1979). The mechanism of opening of the aortic valve. J Thorac Cardiovasc Surg.

[15] Lansac E, Lim HS, Shomura Y, Lim KH, Rice NT, Goetz W (2002). A four-dimensional study of the aortic root
dynamics. Eur J Cardiothorac Surg.

